# Soluble CD14 Enhances the Response of Periodontal Ligament Stem Cells to Toll-Like Receptor 2 Agonists

**DOI:** 10.1155/2019/8127301

**Published:** 2019-04-23

**Authors:** Christian Behm, Alice Blufstein, Johannes Gahn, Nazanin Noroozkhan, Andreas Moritz, Xiaohui Rausch-Fan, Oleh Andrukhov

**Affiliations:** Division of Conservative Dentistry and Periodontology, University Clinic of Dentistry, Medical University of Vienna, Vienna 1090, Austria

## Abstract

Human periodontal ligament stem cells (hPDLSCs) do not express membrane-bound CD14, and their responsiveness to bacterial lipopolysaccharide (LPS) is drastically enhanced by soluble CD14 (sCD14), which is due to the facilitation of the interaction between LPS and Toll-like receptor- (TLR-) 4. Several studies also show that sCD14 enhances the responsiveness of different immune cells to TLR-2, but such effect in hPDLSCs has not been studied so far. In the present study, we investigated for the first time the potential effect of sCD14 on the hPDLSC response to two different TLR-2 agonists, *in vitro*. Primary hPDLSCs were stimulated with synthetic lipopeptide Pam3CSK4 or lipoteichoic acid (LTA) in concentrations 1-1000 ng/ml in the presence/absence of sCD14 (250 ng/ml). Additionally, the effect of different sCD14 concentrations (2.5-250 ng/ml) on the TLR-2 response was determined in Pam3CSK4- or LTA-triggered hPDLSCs. The resulting expression of interleukin- (IL-) 6, chemokine C-X-C motif ligand 8 (CXCL8), and chemokine C-C motif ligand 2 (CCL2) was measured by qPCR and ELISA. The production of IL-6, CXCL8, and CCL2 was gradually increased by both TLR-2 agonists and was significantly enhanced by sCD14. The response of hPDLSCs to low and submaximal concentrations of TLR-2 agonists (1-100 ng/ml) was most effectively enhanced by sCD14. The effect of sCD14 on TLR-2 response in hPDLSCs was concentration-dependent and was already detectable at low sCD14 levels. Our data showed that exogenous sCD14 significantly enhanced the responsiveness of hPDLSCs to TLR-2 agonists and enabled the detection of their small amounts. This effect was already detectable at low sCD14 levels, which are comparable to those in saliva and gingival crevicular fluid. Changes in the local sCD14 level may be considered as a crucial factor influencing the susceptibility of hPDLSCs to different pathogens and thus may contribute to the progression of periodontitis.

## 1. Introduction

Mesenchymal stem cells (MSCs) are multipotent progenitor cells, exhibiting self-renewal potential and an ability to differentiate *in vitro* into multiple cell types [1]. MSCs reside in various dental tissues [2–4], including the periodontal ligament [5]. Human periodontal ligament stem cells (hPDLSCs) are a heterogenous population of fibroblast-like cells [6], which fulfils the minimal criteria for MSCs such as the expression of characteristic surface markers and the multilineage differentiation potential [6, 7]. Similar to other MSCs, hPDLSCs possess immunomodulatory ability and modulate the activity of immune cells by either paracrine mechanisms or direct cell-to-cell contact. hPDLSCs are involved in regulating the processes implicated in periodontal tissue homeostasis, regeneration, and periodontal disease progression [6, 8, 9].

Periodontitis is an inflammatory, multifactorial disease, leading to periodontal tissue destruction and may result in tooth loss in severe cases [10, 11]. It is associated with an impairment of host-microbial homeostasis, leading to an inappropriate, overwhelming immune response [12], and is affected by several risk factors including genetic predisposition [13] and smoking habits [14]. The Gram-negative bacterium *Porphyromonas gingivalis* is most often associated with periodontitis [15, 16] and is currently considered as a keystone pathogen [17]. *P. gingivalis* and its virulence factors, like lipopolysaccharide (LPS) [18, 19] and various lipoproteins [20, 21], induce an inflammatory response in hPDLSCs and influence their immunomodulatory potential.

Bacterial components are recognized by TLRs, a family of pattern recognition receptors of the host cells [21–26]. The activation of TLRs in hPDLSCs results in the production of different inflammatory mediators and is assumed to regulate their immunomodulatory ability [27]. hPDLSCs express different types of TLRs including TLR-2 and TLR-4, which sense lipoproteins and bacterial LPS, respectively [28–30]. Previous studies show that activation of hPDLSCs by *P. gingivalis* LPS leads to the secretion of several potent proinflammatory mediators, like interleukin- (IL-) 1*β*, IL-6, chemokine C-X-X motif ligand 8 (CXCL8), and chemokine C-C motif ligand 2 (CCL2) [18, 19]. In contrast to specialized immune cells, hPDLSCs exhibit a very low reactivity to *P. gingivalis* LPS in concentrations up to 1 *μ*g/ml, reaching a strong activation with only a quite high *P. gingivalis* LPS concentration (10 *μ*g/ml) [18]. The low sensitivity of hPDLSCs to *P. gingivalis* LPS is explained by the lack of membrane-bound CD14 on the hPDLSC surface [9, 19], an important coreceptor of TLR-4 [31, 32]. Our previous study shows that the soluble form of CD14 (sCD14) significantly enhances the response of hPDLSCs to *P. gingivalis* and *Escherichia coli* LPS and allows sensing even low LPS levels in the range of ng/ml [19, 33].

Several studies indicate an association between periodontitis progression and TLR-2 (reviewed in [34]). Lipoproteins of different bacteria, including *P. gingivalis*, are the main ligands of TLR-2 [20, 21, 35]. A study of Morandini et al. shows that lipoprotein-induced expression of IL-6 and CXCL8 is significantly decreased in TLR-2-silenced periodontal ligament fibroblasts [21]. A recent study of our group shows that the synthetic TLR-2 agonist Pam3CSK4, a triacylated lipoprotein, induces a significantly higher inflammatory response in hPDLSCs than bacterial LPS [19].

Previous studies on distinct immune cells suggest that sCD14 might enhance the TLR-2 activation in the presence of an appropriate agonist [36–39]. Ranoa et al. demonstrates an involvement of sCD14 in the interaction of TLR-2 with synthetic triacylated lipopeptides, making cells susceptible for lipopeptides even on the nanogram range [38]. Further, Nakata et al. shows that CD14 directly binds triacylated lipopeptides, without binding TLR-2 [36]. Additionally, other studies also show a CD14-dependent induction of the cytokine synthesis in T cells and monocytes after stimulation with TLR-2 agonist lipoteichoic acid (LTA) [37, 40]. To the best of our knowledge, the potential of sCD14 to modulate the activation of TLR-2 in hPDLSCs is not known to date. Therefore, in the present study, we investigated the effect of sCD14 on the response of hPDLSCs to TLR-2 agonists. Particularly, we measured the expression of IL-6, CXCL8, and CCL2 in hPDLSCs upon stimulation with different concentrations of the synthetic triacylated lipoprotein Pam3CSK4 or LTA in the presence/absence of a single sCD14 concentration. Further, we tested the dependency of the hPDLSC response to a submaximal response to TLR-2 agonists on different sCD14 concentrations.

## 2. Material and Methods

### 2.1. Cell Culture

Third molars from five different periodontally healthy patients were extracted due to orthodontic reasons and used to isolate primary hPDLSCs, as described in our previous study [19]. All patients were informed and gave their written consent before the surgical procedure. The study protocol was approved by the Ethics Committee of the Medical University of Vienna. All procedures were performed according to the “Good Scientific Practice” guidelines of the Medical University of Vienna and the Declaration of Helsinki. Primary hPDLSCs were cultured under humidified conditions in Dulbecco's modified Eagle's medium (DMEM, Sigma-Aldrich, St. Louis, USA), supplemented with 10% fetal bovine serum (FBS, Gibco, Carlsbad, USA), 1% penicillin, and streptomycin (P/S, Gibco, Carlsbad, USA). Cells between the 3rd and 7th culture passages were used for the experiments. Cell surface marker expression of mesenchymal stem cells (CD29, CD90, CD105, and CD146) and of hematopoietic cells (CD14, CD31, CD34, and CD45) was analysed to verify minimal MSC criteria in isolated hPDLSCs [19].

### 2.2. Stimulation Protocol

Primary hPDLSCs were seeded in 24-well plates at a density of 5 × 10^4^ cells per well, in 0.5 ml DMEM, supplemented with 1% P/S and 10% FBS. In the first series of experiments, the effect of sCD14 in a constant concentration of 250 ng/ml on the response of hPDLSCs to different concentrations of TLR-2 agonists was investigated. The sCD14 concentration was chosen due to our experience from a previous study [19]. Cells were stimulated with either TLR-2/1 agonist Pam3CSK4 (InvivoGen, San Diego, USA) or TLR-2 agonist lipoteichoic acid (LTA, InvivoGen, San Diego, USA) 24 hours after seeding. Stimulation was done with different concentrations (1, 10, 100, and 1000 ng/ml) of TLR-2 agonists in the presence/absence of exogenous sCD14 (Sigma-Aldrich, St. Louis, USA). In the second series of experiments, the impact of different sCD14 concentrations on the hPDLSC response to constant concentrations of TLR-2 agonists was examined. In these experiments, the cells were treated with submaximal concentrations (10 ng/ml) of either Pam3CSK4 or LTA in the presence of sCD14 at concentrations 2.5, 10, 25, 100, and 250 ng/ml. All stimulations were performed in duplicates and in FBS-free DMEM, supplemented with 1% P/S. After 24 hours of stimulation, IL-6, CXCL8, and CCL2 gene expression levels were measured by quantitative polymerase chain reaction (qPCR), and the levels of corresponding proteins in the conditioned media were determined using the enzyme-linked immunosorbent assay (ELISA).

### 2.3. Quantitative PCR

The TaqMan Gene Expression Cells-to-CT kit (Applied Biosystems, Foster City, USA) was used for cell lysis, mRNA extraction, reverse transcription into cDNA, and qPCR according to the manufacturer's protocol. Reverse transcription was conducted on the Primus 96 advanced thermocycler (PeqLab/VWR, Darmstadt, Germany) using the following settings: 37°C for 1 hour and 95°C for 5 minutes for enzyme deactivation followed by 4°C. qPCR was performed using the ABI StepOnePlus device (Applied Biosystems, Foster City, USA) with the following thermocycler settings: 10 minutes at 1 × 95°C followed by 50 × 15 seconds at 95°C and 1 minute at 60°C. For amplifying the target genes, the following TaqMan Gene Expression Assays (Applied Biosystems, Foster City, USA) were used: IL-6, Hs00985639_m1; CXCL8, Hs00174103_m1; CCL2, Hs00234140_m1; and GAPDH Hs99999905. qPCR was performed in paired reactions, followed by specifying the Ct value for each sample. Gene expression levels were quantified using the 2^−ΔΔCt^ method by the formula

(1) $$\begin{array}{c} \Delta \Delta \text{Ct}={\left({\text{Ct}}^{\text{target}}–{\text{Ct}}^{\text{GAPDH}}\right)}_{\text{sample}}–{\left({\text{Ct}}^{\text{target}}–{\text{Ct}}^{\text{GAPDH}}\right)}_{\text{control}}. \end{array}$$

The *n*-fold expression of the target genes compared to the corresponding untreated control was determined. GAPDH served as endogenous reference.

### 2.4. Enzyme-Linked Immunosorbent Assay

For determining IL-6, CXCL8, and CCL2 protein levels in the conditioned media, ELISA Ready-Set-Go! Kits (eBioscience, Waltham, USA) were used, according to the manufacturer's protocol. ELISAs were performed in duplicates, followed by measuring optical density at 450 nm. Measured absorbance values were plotted against the corresponding standard curves, determining the appropriate protein concentrations.

### 2.5. Statistical Analysis

The statistical program SPSS 24.0 (IBM, Armonk, USA) was used for all statistical analysis. The Friedman test followed by the Wilcoxon test for pairwise comparison was used. Differences with *p* values < 0.05 were considered as statistically significant.

## 3. Results

### 3.1. Effect of sCD14 on the hPDLSC Response to TLR-2 Agonist Pam3CSK4

**Figure 1:**
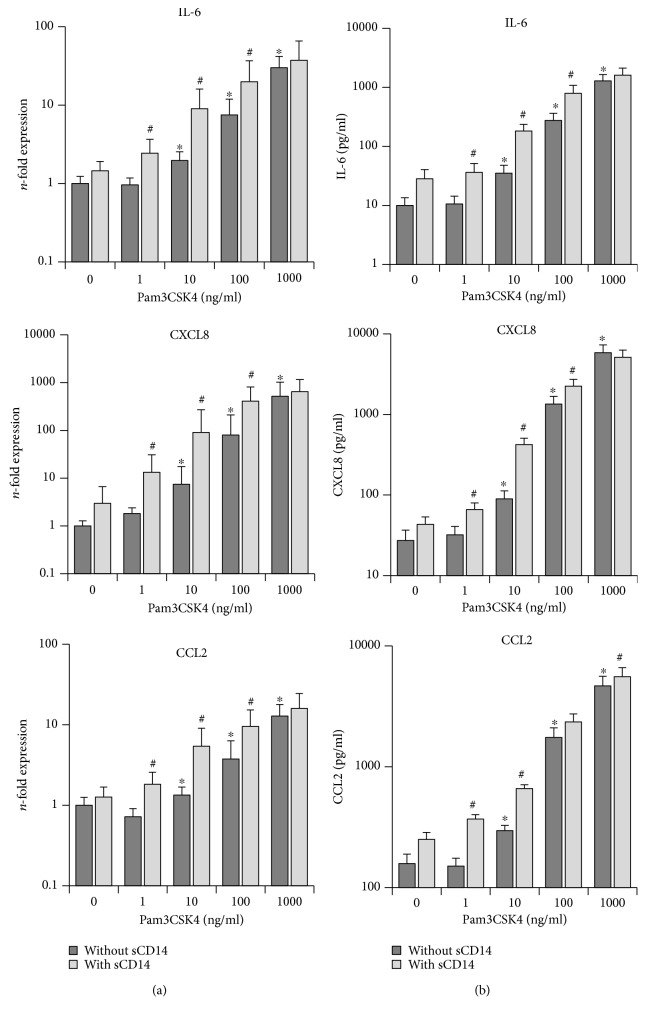
Effect of sCD14 on the hPDLSC response to TLR-2 agonist Pam3CSK4. Primary hPDLSCs were stimulated with different Pam3CSK4 concentrations (1-1000 ng/ml), in the presence/absence of sCD14 (250 ng/ml). Untreated cells served as the control. After 24 hours of incubation, IL-6, CXCL8, and CCL2 gene expression levels (a) were measured using qPCR. The *y*-axis shows the *n*-fold expression of the target genes compared to the unstimulated control. The corresponding protein levels in conditioned media (b) were determined by ELISA. All data are presented as mean ± s.e.m. from five independent experiments using cells from five different donors. ^∗^*p* value < 0.05 compared to control. #*p* value < 0.05 compared to the appropriate group stimulated in the absence of sCD14.

Gene and protein expression levels of IL-6, CXCL8, and CCL2 in primary hPDLSCs, stimulated with different Pam3CSK4 concentrations in the presence/absence of sCD14 (250 ng/ml), are shown in Figure 1. In the absence of sCD14, Pam3CSK4 induced a concentration-dependent increase in IL-6, CXCL8, and CCL2 gene expression levels. The highest response was observed at 1000 ng/ml Pam3CSK4. Stimulation with 10 to 1000 ng/ml Pam3CSK4 led to a significant increase in IL-6, CXCL8, and CCL2 gene expressions. Exogenous sCD14 significantly enhanced the expressions of IL-6, CXCL8, and CCL2 at submaximal Pam3CSK4 concentrations (1-100 ng/ml). The response to the highest Pam3CSK4 concentration (1000 ng/ml) was not significantly affected by exogenous sCD14.

Protein measurements with ELISA showed that in the absence of sCD14, IL-6, CXCL8, and CCL2 protein levels were significantly increased after stimulation with Pam3CSK4 starting from 10 ng/ml in a concentration-dependent manner. The presence of sCD14 during stimulation resulted in significantly enhanced levels of all proteins in response to all tested Pam3CSK4 concentrations excepting the highest one (1000 ng/ml).

### 3.2. Effect of sCD14 on the hPDLSC Response to TLR-2 Agonist LTA

**Figure 2:**
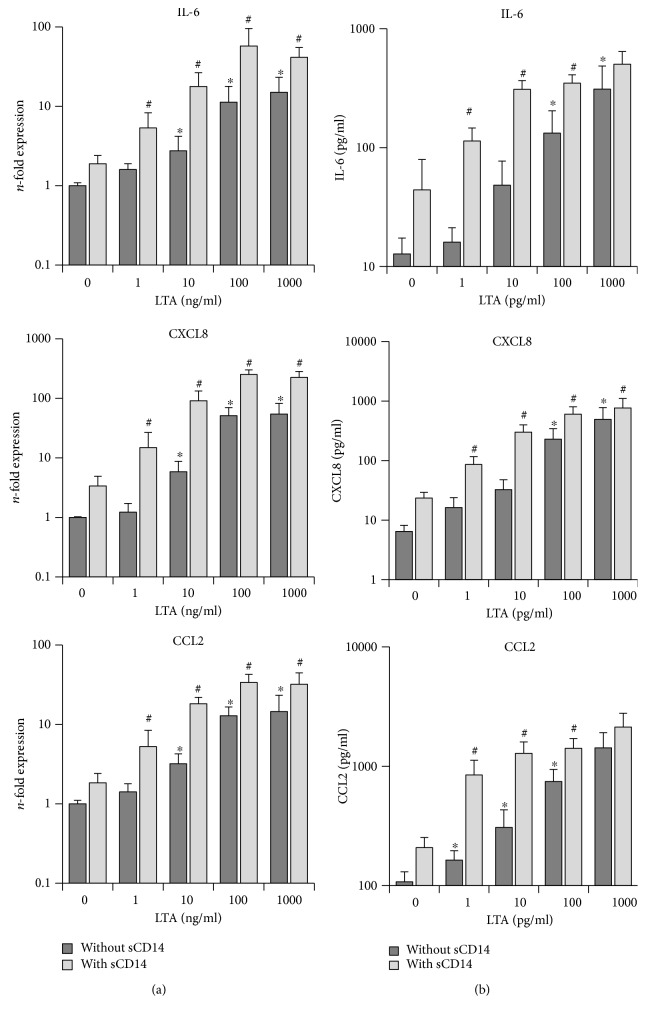
Effect of sCD14 on the hPDLSC response to TLR-2 agonist LTA. Primary hPDLSCs were stimulated with different LTA concentrations (1-1000 ng/ml), in the presence/absence of sCD14 (250 ng/ml). Untreated cells served as the control. After 24 hours of incubation, IL-6, CXCL8, and CCL2 gene expression levels (a) were measured using qPCR. The *y*-axis shows the *n*-fold expression of the target genes compared to the unstimulated control. The corresponding protein levels in conditioned media (b) were determined by ELISA. All data are presented as mean ± s.e.m. from five independent experiments using cells from five different donors. ^∗^*p* value < 0.05 compared to the control. #*p* value < 0.05 compared to the appropriate group stimulated in the absence of sCD14.

Gene and protein expression levels of IL-6, CXCL8, and CCL2 in primary hPDLSCs, stimulated with different LTA concentrations in the presence/absence of sCD14 (250 ng/ml), are shown in Figure 2. In the absence of sCD14, LTA induced a concentration-dependent increase in IL-6, CXCL8, and CCL2 gene expression levels. The highest response was observed at 1000 ng/ml LTA. A significant increase in the expression of all three target genes was observed after stimulation from 10 to 1000 ng/ml LTA. Adding the exogenous sCD14 during stimulation significantly enhanced the expression levels of all three target genes in response to all tested LTA concentrations.

As measured by ELISA, in the absence of sCD14, IL-6, CXCL8, and CCL2 protein levels were significantly increased after stimulation with LTA in a dose-dependent manner. A significant increase started at 100 ng/ml for IL-6 and CXCL8 and at 1 ng/ml for CCL2. The presence of exogenous sCD14 during stimulation resulted in significantly enhanced levels of IL-6 and CCL2 in response to all tested LTA concentrations excepting the highest one. Additionally, sCD14 significantly increased CXCL8 protein production induced by all tested LTA concentrations.

### 3.3. Effect of Different sCD14 Concentrations on the hPDLSC Response to Submaximal Concentration of Pam3CSK4

**Figure 3:**
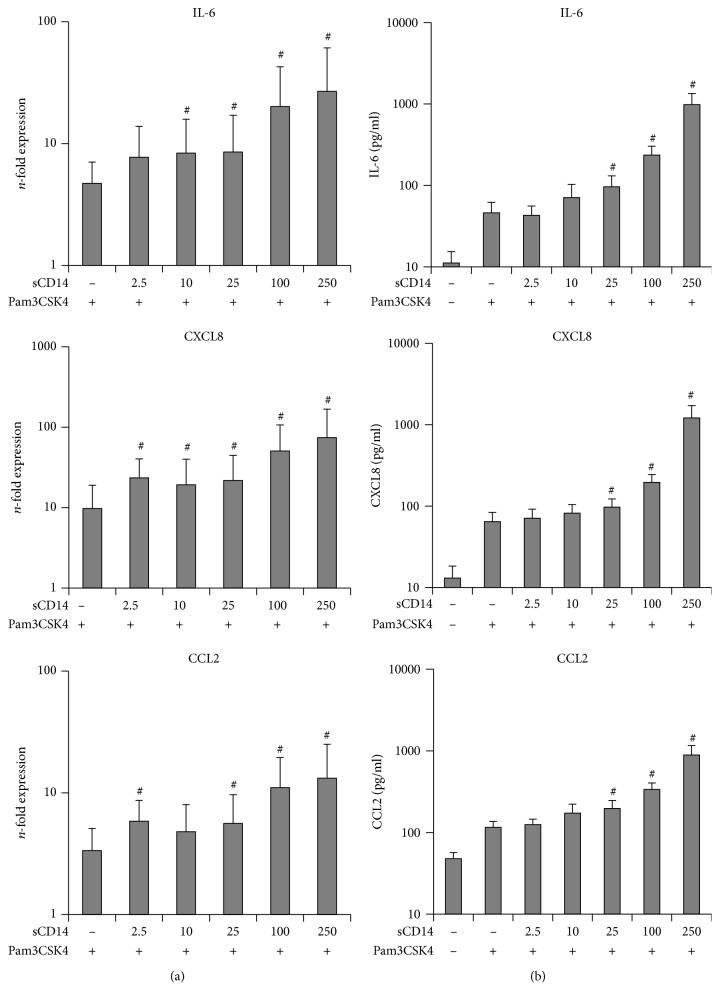
Effect of different sCD14 concentrations on the hPDLSC response to submaximal Pam3CSK4 concentration. Primary hPDLSCs were stimulated with Pam3CSK4 at submaximal concentration (10 ng/ml) and different concentrations of sCD14 (2.5–250 ng/ml). Untreated cells served as the control. After 24 hours of incubation, IL-6, CXCL8, and CCL2 gene expression levels (a) were determined using qPCR. The *y*-axis shows the *n*-fold expression of the target genes compared to the unstimulated control. The corresponding protein levels in conditioned media (b) were determined by ELISA. All data are presented as mean ± s.e.m. from five independent experiments using cells from five different donors. #*p* value < 0.05 compared to cells stimulated with Pam3CSK4 in the absence of sCD14.

The dependency of the hPDLSC response to submaximal Pam3CSK4 concentration (10 ng/ml) on different sCD14 levels is shown in Figure 3. Stimulation of hPDLSCs with Pam3CSK4 in the absence of sCD14 resulted in increased IL-6, CXCL8, and CCL2 gene expression levels. This response to Pam3CSK4 was increased by sCD14 in a concentration-dependent manner. A significant increase was observed starting from 10 ng/ml sCD14 for IL-6 and from 2.5 ng/ml for CXCL8 and CCL2.

In the absence of sCD14, 10 ng/ml Pam3CSK4 already induced protein expression of IL-6, CXCL8, and CCL2 in a concentration-dependent manner. A significant increase of all three investigated proteins was observed starting from sCD14 concentration as low as 25 ng/ml.

### 3.4. Effect of Different sCD14 Concentrations on the hPDLSC Response to Submaximal Concentration of LTA

**Figure 4:**
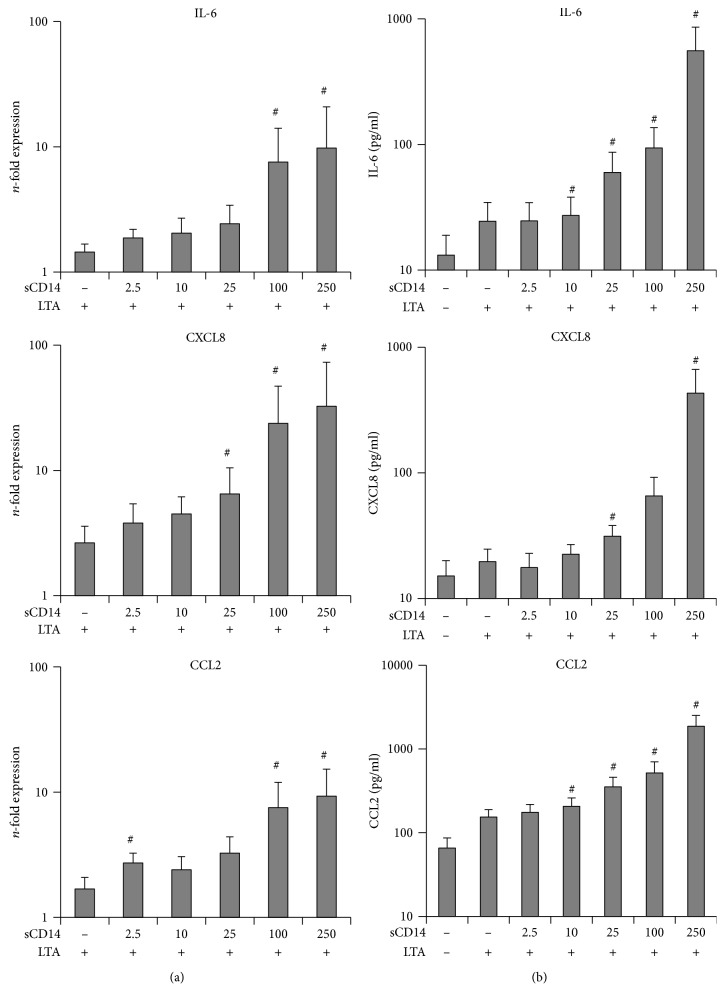
Effect of different sCD14 levels on the hPDLSC response to submaximal LTA concentration. Primary hPDLSCs were stimulated with LTA at submaximal concentration (10 ng/ml) and different concentrations of sCD14 (2.5-250 ng/ml). Untreated cells served as the control. After 24 hours of incubation, IL-6, CXCL8, and CCL2 gene expression levels (a) were measured using qPCR. The *y*-axis shows the *n*-fold expression of the target genes compared to the unstimulated control. The corresponding protein levels in conditioned media (b) were determined by ELISA. All data are presented as mean ± s.e.m. from five independent experiments using cells from five different donors. #*p* value < 0.05 compared to cells stimulated with LTA in the absence of sCD14.

The dependency of hPDLSC response to submaximal LTA concentration (10 ng/ml) on different sCD14 levels is shown in Figure 4. LTA treatment of hPDLSCs in the absence of sCD14 caused an increase in gene expressions of IL-6, CXCL8, and CCL2. This response to LTA was increased by sCD14 in a concentration-dependent manner, showing significances at higher sCD14 levels for all three genes. Gene expression of CCL2 was already significantly increased at the lowest tested sCD14 level (2.5 ng/ml).

Protein expressions of all three targets were increased upon LTA stimulation and were further enhanced by exogenous sCD14 in a concentration-dependent manner. Statistically significant differences were observed starting from 10 ng/ml sCD14 for IL-6 and CCL2 and from 25 ng/ml sCD14 for CXCL8.

### 3.5. Response of hPDLSCs to Exogenous sCD14

**Figure 5:**
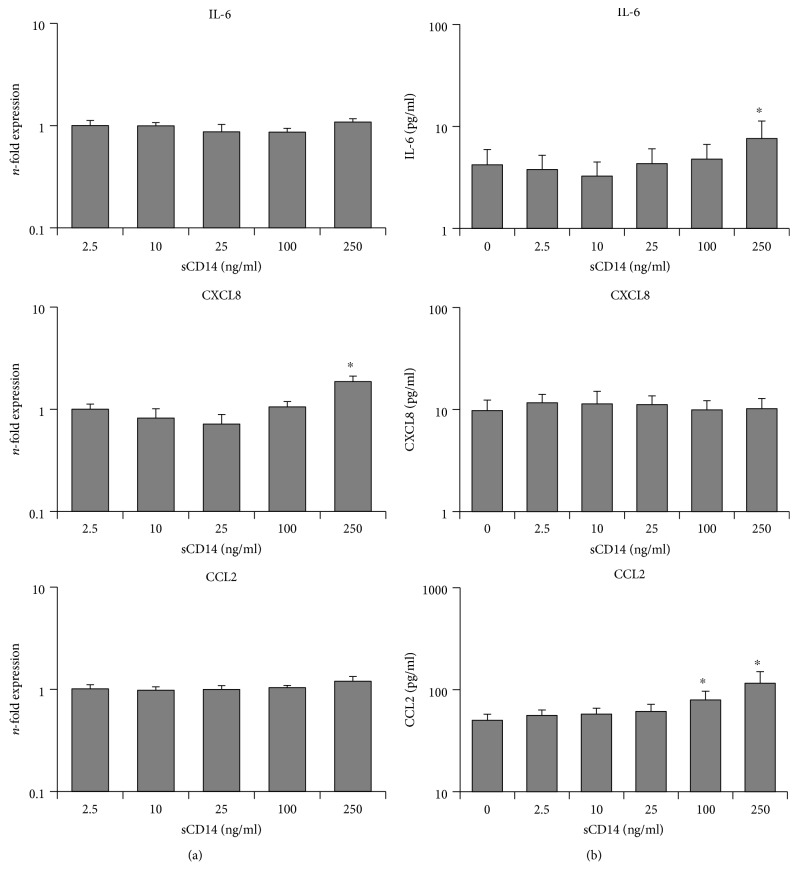
Response of hPDLSCs to different sCD14 concentrations. Primary hPDLSCs were stimulated for 24 hours with different sCD14 concentrations (2.5 ng/ml–250 ng/ml) in the absence of TLR-2 agonists. Untreated cells served as the control. After 24 hours of incubation, IL-6, CXCL8, and CCL2 gene expression levels (a) were measured using qPCR. The *y*-axis shows the *n*-fold expression of the target genes compared to the unstimulated control. The corresponding protein levels in conditioned media (b) were determined by ELISA. All data are presented as mean ± s.e.m. from five independent experiments using cells from five different donors. ^∗^*p* value < 0.05 compared to the control.

Figure 5 shows the response of hPDLSCs to exogenous sCD14 without any TLR-2 agonist. Under these conditions, low concentrations of exogenous sCD14 have no significant effect on IL-6, CXCL8, and CCL2 expressions, on gene and protein levels. However, significances were observed only for the highest tested sCD14 levels (250 ng/ml) for IL-6 protein and CXCL8 gene expressions as wells as starting at 100-250 ng/ml for CCL2 protein expression.

## 4. Discussion

In our previous study, we showed a significant sCD14-mediated increase in the bacterial LPS- induced TLR-4 response in hPDLSCs [19]. Although several studies on different immune cells showed an impact of sCD14 on TLR-2 [36–39], the contribution of sCD14 on the TLR-2 response in hPDLSCs has not been investigated so far. Hence, we investigated for the first time *in vitro* the potential impact of sCD14 on Pam3CSK4- or LTA-induced TLR-2 response in hPDLSCs. Our data demonstrated that exogenous sCD14 substantially enhances the production of inflammatory mediators by hPDLSCs in response to stimulation even with small amounts of TLR-2 agonists. This finding further indicates an important role of sCD14 in the host inflammatory response to different bacterial components.

The production of the inflammatory mediators IL-6, CXCL8, and CCL2 was substantially increased by exogenous sCD14 in response to both tested TLR-2 agonists. The enhancement of the response was observed for all tested Pam3CSK4 and LTA concentrations excepting the highest tested Pam3CSK4 concentration of 1000 ng/ml. The presence of exogenous sCD14 allowed hPDLSCs to sense different TLR-2 agonists at very low concentrations, starting from 1 ng/ml, and thus, might sense very low amounts of bacterial pathogens. Such situation is imaginable immediately after epithelial barrier destruction and bacterial invasion into periodontal tissue. Production of different inflammatory mediators by hPDLSCs under these conditions will substantially contribute to the further host inflammatory response and disease progression.

In our study, we focused on the expression of three proteins, IL-6, CXCL8, and CCL2, which are thought to play an essential role in periodontal tissue inflammation and are usually regarded as the factors which enhance the inflammatory response in periodontitis [41, 42]. IL-6 is an important proinflammatory cytokine, involved in the acute inflammation phase and bone resorption [41]. CXCL8 and CCL2 are both chemokines, attracting neutrophils and macrophages, respectively, to the site of inflammation, promoting acute inflammation [42, 43]. However, studies of the last years suggest that besides classical proinflammatory effects, these proteins play some anti-inflammatory roles and/or contribute to periodontal tissue homeostasis. Particularly, there are increasing evidences that IL-6 is involved in MSC-dependent suppression of T cell proliferation. There are also evidences that IL-6 suppresses neutrophil apoptosis and respiratory burst and facilitates regulatory dendritic cell and anti-inflammatory macrophage formation [44–47]. Lower CXCL8 levels are thought to diminish neutrophil recruitment to the periodontal pocket, which may lead to overgrowth of some pathogenic microorganisms.

Based on this dual role of all investigated mediators, it is difficult to assess the exact role of local sCD14 levels and the enhancement of TLR-2-induced response by sCD14. It might on the one hand promote the inflammation response and on the other hand contribute to the maintenance of periodontal tissue homeostasis. The dual role of the immune response should be also noted, which on the one hand is aimed at eliminating pathogens and on the other hand leads to collateral tissue damages [48]. Hence, the physiological functions and the exact role in periodontal tissue inflammation of IL-6, CXCL8, and CCL2, produced by hPDLSCs in response to different virulence factors, need to be further investigated.

Our data showed a significant effect of sCD14 on the TLR-2 response in hPDLSCs regardless of the TLR-2 agonist. This may occur obviously by facilitating the interaction between lipoproteins and TLR-2 on the hPDLSC surface by sCD14 [36–38, 40]. However, the hPDLSC response to 1000 ng/ml Pam3CSK4 was not further enhanced by sCD14, which was also observed in our previous study [19]. This finding can be explained by the fact that Pam3CSK4 at this concentration induces a maximal TLR-2 response and that TLR-2 signalling seems to be fully activated even in the absence of sCD14. This observation is in line with other studies, exhibiting CD14 as critical for the response to low LPS doses and less important for high LPS doses [49] and that sCD14 makes immune cells more susceptible to triacetylated lipopeptides on the nanogram level [38].

In contrast to Pam3CSK4, sCD14 significantly enhanced the hPDLSC response to LTA at 1000 ng/ml. However, it should be noted that in the absence of sCD14, the production of different proinflammatory mediators by hPDLSCs in response to 1000 ng/ml LTA was up to 90% lower than that induced by similar concentration of Pam3CSK4. Therefore, it can be assumed that LTA at 1000 ng/ml did not induce a maximal activation of TLR-2-dependent response, which can be further increased by adding exogenous sCD14. The differences in the hPDLSC response might be explained by the distinct chemical nature of the two used TLR-2 agonists and the resulting different activation mechanisms [36–38, 40, 50].

The molecular mechanisms of the cell response activation by TLR-2 agonists are differently discussed in the literature. Triacylated lipoprotein Pam3CSK4 coordinately binds to TLR-2 as well as to TLR-1, leading to a heterodimerization of these two receptors and resulting in the formation of a stable ternary signalling complex, consisting of TLR-2, TLR-1, and triacetylated lipoprotein [36, 38, 50]. sCD14 as well as lipopolysaccharide-binding protein (LBP) sensitize cells to lipoproteins [51, 52] by disaggregating lipoproteins and delivering monomeric lipoproteins to the receptors [36, 38]. In contrast to the membrane-bound CD14, which is physically associated with the TLR heterodimer [53], sCD14 facilitates the formation of the ternary signalling complex without being a part of the complex itself. A study of Ranoa et al. suggests that during agonist delivery to the receptors, sCD14 stably interacts with TLR-1 but is replaced by TLR-2 during ternary complex formation [38]. Concerning LTA, several functional studies show an LTA-activated cellular response through TLR-2 recognition without the involvement of other TLRs but in the presence of sCD14 [37, 40, 54]. However, the knowledge about the precise interactions of involved proteins is limited. One study demonstrates the complex formation of LTA with LBP and catalytic transfer of LTA to sCD14 through LBP, resulting in the formation of a LTA-sCD14 complex [37]. Although both TLR-2 agonists seem to differ in the TLR activation mechanism, they cause NF-*κ*B translocation into the nucleus and further the expression of proinflammatory cytokines [37, 55].

We further investigated the dependency of the hPDLSC response to submaximal concentrations of TLR-2 agonists on different local sCD14 levels. We found a clear dose-dependent increase of the TLR-2 response from the lowest to the highest sCD14 concentrations. On the protein level, a significant increase in hPDLSC response to submaximal concentrations of TLR-2 agonists was induced by 25 ng/ml of sCD14, which is a rather low level compared to those detected in physiological fluids. Particularly, the local sCD14 concentration in gingival crevicular fluid and blood serum is in *μ*g/ml [56, 57]. Since the effect of sCD14 levels on the hPDLSC response to both TLR-2 agonists and bacterial LPS [19] is concentration-dependent, we suggest that alterations of local sCD14 levels might play an important role in periodontitis through sensitizing hPDLSCs to periodontal pathogens. For example, an increase in sCD14 will facilitate the recognition of periodontal bacteria-associated lipoproteins of LPS by hPDLSCs. This enhances the production of diverse inflammatory mediators, including IL-6, CXCL8, and CCL2, which may contribute to the overwhelming immune response and consequently to progression of periodontitis. This assumption is supported by different clinical studies, which mostly show a positive association between sCD14 levels and periodontal disease [57–59]. Particularly, Isaza-Guzmán et al. detected significant higher sCD14 levels in the saliva of periodontitis patients compared to healthy controls [58], whereas other studies also showed a significantly higher sCD14 concentrations in the serum of subjects with chronic periodontitis [57, 59]. Additionally, a positive correlation between sCD14 saliva levels and different clinical parameters of periodontitis severity was demonstrated [58]. Interestingly, Jin and Darveau exhibited a negative correlation between sCD14 levels in gingival crevicular fluids and the number and depth of periodontal pockets in patients suffering from periodontitis [56]. These inconsistencies, concerning the relationship between local sCD14 levels and periodontitis severity, may be explained by the dual nature of the immunomodulatory properties of hPDLSCs. On the one hand, hPDLSCs produce proinflammatory mediators under certain conditions, promoting the inflammatory response. On the other hand, hPDLSCs are known to produce immunosuppressive proteins, which diminish the local inflammatory response [60]. Regulation of the immunomodulatory activity of hPDLSCs by TLR activation may effect periodontal disease progression [19, 27]. This assumption is supported by studies, showing an impaired immunomodulation of hPDLSCs in periodontitis patients [61, 62].

It seems that there are some differences in the affinity of sCD14 to different TLRs in hPDLSCs. In our previous study, we show that the maximal effect of sCD14 on the TLR-4 response to bacterial LPS is already achieved at concentrations of 25 ng/ml [19]. In contrast, in the present study, a gradual increase in hPDLSC response to both TLR-2 agonists was observed with a gradual increase of the sCD14 concentration from 2.5 to 250 ng/ml. Although sCD14 facilitates the formation of a stable ternary signalling complex for both TLRs, without being a part of the complex itself [36, 38, 63], the activation mechanisms of both TLRs differ from each other, due to the different natures of their agonists or the involvement of the MD-2 protein in the TLR-4 signalling complex [64]. These differences may possibly explain the higher sensitivity of the TLR-4 response to sCD14 than TLR-2.

We further investigated a potential influence of sCD14 on the expression of inflammatory markers in hPDLSCs in the absence of TLR-2 agonists. Surprisingly, we found a significant increase in the secretion of IL-6 and CCL2 and a significant increase in the expression of CXCL8 at the highest used sCD14 concentrations. However, the expression levels, triggered solely by sCD14, were rather negligible compared with its effect on Pam3CSK4- or LTA-induced responses. Therefore, we conclude that the enhancement of the TLR-2 responses by sCD14 is due to its interaction with the TLR-2 receptor. Additionally, the stable interaction of sCD14 with TLR-1 until the heterodimerization with TLR-2 [38] may influence the expression of inflammatory markers, at least at high sCD14 concentrations.

## 5. Conclusion

In conclusion, our study shows that sCD14 enhances the response of hPDLSCs to submaximal concentrations of TLR-2 agonists in a concentration-dependent manner. The stimulating effect was detectable at sCD14 levels, which are comparable to those in saliva and gingival crevicular fluid. The presence of sCD14 sensitizes hPDLSCs to bacterial pathogens and enables their response even to low amounts of TLR-2 agonists in the range of few nanograms per millilitres. We suggest that different local sCD14 levels may affect the strength of TLR-2-mediated immune response in hPDLSCs, leading to a stronger IL-6, CXCL8, and CCL2 production at higher sCD14 levels. These higher proinflammatory cytokine levels may contribute to the overwhelming immune response and consequently affect the development of periodontitis.

## Data Availability

The data used to support the findings of this study are available from the corresponding author upon request.
